# Metabolic signatures in gastroenteropancreatic neuroendocrine neoplasms: unraveling diagnostic and prognostic insights

**DOI:** 10.3389/fendo.2025.1676021

**Published:** 2025-12-11

**Authors:** Reydson Alcides de Lima-Souza, Tayná Figueiredo Maciel, Moisés Willian Aparecido Gonçalves, Fernanda Cristina Poscai Ribeiro, Rodrigo A. Maioral, João Figueira Scarini, Heloisa P Soraes, Gary Chris Fillmore, Erika Said Abu Egal

**Affiliations:** 1 Biorepository and Molecular Pathology, Huntsman Cancer Institute, University of Utah (UU), Salt Lake City, UT, United States; 2 Department of Pathology, School of Medical Sciences, University of Campinas (UNICAMP), Campinas, São Paulo, Brazil; 3 Department of Oral Diagnosis, Piracicaba Dental School, University of Campinas (UNICAMP), Piracicaba, São Paulo, Brazil; 4 Department of Internal Medicine, University of Western São Paulo (UNOESTE), Guarujá, São Paulo, Brazil; 5 Department of Oncology, Huntsman Cancer Institute, University of Utah (UU), Salt Lake City, UT, United States

**Keywords:** neuroendocrine neoplasms, gastroenteropancreatic neoplasms, metabolic reprogramming, metabolic biomarkers, diagnosis

## Abstract

Gastroenteropancreatic neuroendocrine neoplasms (GEP-NENs) are a heterogeneous group of tumors characterized by diverse biological behaviors and variable clinical outcomes. Recent advances have highlighted the important role of metabolic reprogramming in tumorigenesis, progression, and therapeutic resistance in GEP-NENs. In this review, we synthesize the current evidence on metabolic biomarkers and altered metabolic pathways—particularly those involving glucose, lipid, and amino acid metabolism. Key biomarkers such as GLUT-1, FASN, and enzymes involved in ferroptosis, cholesterol biosynthesis, and amino acid catabolism demonstrate strong associations with tumor aggressiveness, hypoxia, and mTOR signaling. Moreover, metabolomic profiling and functional studies suggest that metabolic markers may inform prognosis and predict response to targeted therapies such as Everolimus. Although promising, the clinical translation of these markers is still limited and requires further validation in large, subtype-specific cohorts. Our findings highlight the importance of integrating metabolic profiling into the diagnostic and therapeutic landscape of GEP-NENs. Future research should prioritize biomarker standardization, multi-omics integration, and the development of metabolism-based therapeutic strategies tailored to tumor subtype and differentiation grade.

## 1 Introduction

Gastroenteropancreatic neuroendocrine neoplasms (GEP-NENs) are a subset of neuroendocrine neoplasms (NENs) that arise in the stomach, small intestine, appendix, and pancreas (1). Over the past few decades, the global incidence of these tumors has increased significantly (2, 3). However, due to their typically mild and nonspecific symptoms, diagnosis is often delayed, with many cases only being detected at advanced stages (4). Thus, the tumor stage and the presence of metastases become crucial prognostic factors, significantly influencing patient survival (5).

Dysregulated metabolism is a hallmark of cancer, leading to the accumulation of aberrant metabolites that reflect both tumor activity and the underlying biological state (6). Recently, metabolites have gained increasing attention as promising biomarkers for early detection, prognostic assessment, and therapeutic monitoring. In GEP-NENs, several metabolic alterations have been identified that reflect tumor behavior and biology, offering valuable insights into potential targeted strategies (6–8).

Advances in analytical technologies have enabled comprehensive profiling of tumor-associated metabolites, thus enhancing our understanding of tumor metabolism and supporting the development of more precise, personalized therapeutic approaches (9). Despite this progress, the clinical translation of these findings remains a challenge, underscoring the need for continued research in this area (9).

This review synthesizes and critically evaluates current research to provide a deeper understanding of how metabolites profiling can bridge experimental discoveries and clinical practice in cancer diagnosis, management, and prognosis, with a particular focus on GEP-NENs.

## 2 NENs and pancreatic neuroendocrine neoplasms

NENs are a rare and heterogeneous group of malignant tumors that arise from cells of the neuroendocrine system and are mainly derived from two cell types: epithelial neuroendocrine cells and neuronal/para-neuronal cells (1). These cells are characterized by their ability to produce, store, and secrete hormones and other biologically active substances, giving NENs a wide range of clinical presentations and biological behaviors (10, 11).

Among the NENs, those of the GEP represent a significant proportion, predominantly affecting organs such as the stomach, small intestine, colon, rectum, and pancreas (12). GEP-NENs show considerable variability in their clinical and biological behavior, ranging from indolent, slow-growing forms to highly aggressive tumors with great potential for local invasion and distant metastasis (1, 10).

In recent decades, the understanding of these neoplasms has evolved significantly, driven by advances in molecular biology, diagnostic imaging, histopathologic techniques, and targeted therapies. These advances have contributed to better prognostic stratification and the development of more individualized therapeutic strategies, allowing a more precise and effective approach to the management of patients with GEP-NENs (10, 12).

### 2.1 Etiopathogenesis

The etiology of NENs is unknown. Hereditary genetic defects, including multiple endocrine neoplasia type 1, von Hippel-Lindau syndrome, and neurofibromatosis type 1, are associated with NETs of the thorax and upper digestive tract (1).

Known risk factors include a family history of cancer, advanced age, high body mass index, and site-specific risk factors common to non-neuroendocrine cancers, including smoking and alcohol consumption (13–15).

The pathogenesis is unknown, except for the enterochromaffin-like cell neuroendocrine tumors (NET) of the stomach, for which hypergastrinemia is the obligate promoting factor (1, 14, 15). Associated conditions include chronic atrophic gastritis (autoimmune or Helicobacter pylori infection-related), and multiple endocrine neoplasia type 1 (Zollinger-Ellison syndrome) may be involved (1, 13).

### 2.2 Classification

NEN is used as an umbrella term for all neoplasms that originate from cells of the neuroendocrine system (**Table 1**). NENs are classified based on their morphological differentiation: either well-differentiated neuroendocrine tumors (NETs) or poorly differentiated neuroendocrine carcinomas (NECs). These categories have distinct clinical behaviors and prognostic implications (1, 16). NETs are well-differentiated tumors with preserved cellular architecture and a relatively organized histologic pattern. Despite their homogeneous morphology, these tumors exhibit variable biological behavior, which is why they are stratified into three grades according to the rate of cell proliferation, using the Ki-67 index as the main parameter: NET grade 1 (G1), with Ki-67 <3%; NET grade 2 (G2), with Ki-67 between 3% and 20%; and NET grade 3 (G3), with Ki-67 >20% (1, 16).

**Table 1:** Classification of neuroendocrine neoplasms.

Term	Differentiation	Grade	Ki-67	Behavior
Neuroendocrine neoplasms	Any	Any	Any	Any
Neuroendocrine tumors	Well	Low-high	3-20%	Indolent or agressive
Neuroendocrine carcinoma	Poorly	High	>20%	Agressive
Mixed neuroendocrine–non-neuroendocrine neoplasms	Any	Any	Any	Any

It is worth remembering that the current classification recognizes that some well-differentiated tumors may exhibit a more aggressive clinical behavior while retaining the morphologic features that are typical of NET (1).

Alternately, NECs are high-grade, poorly differentiated tumors with highly aggressive behavior and rapid growth. They have a high proliferative rate, usually with a Ki-67 index greater than 20% and are divided into two main morphologic subtypes: small-cell neuroendocrine carcinoma and large-cell neuroendocrine carcinoma. Although the concept of progression from NET to NEC has not been fully elucidated, the distinction between these two subgroups is essential as it directly influences both prognosis and choice of therapeutic strategies (1, 17, 18).

In addition to morphological differentiation and proliferative index, another fundamental aspect in the characterization of NENs is their secretory potential. NENs, especially well-differentiated NETs, can produce, store, and secrete biologically active substances, such as peptide hormones, biogenic amines, and other mediators. Based on this functional profile, NEN are classified as functional and non-functional. Functioning tumors secrete hormones in sufficient quantities to cause specific clinical manifestations, whereas non-functioning tumors, on the other hand, do not secrete hormones at clinically significant levels and therefore are generally not associated with clinical hormonal syndromes (1, 12).

In this context, immunohistochemical evaluation plays a central role in the diagnosis of NENs, with chromogranin A and synaptophysin being the most widely used markers, as they reflect the presence of secretory structures typical of neuroendocrine cells (1, 19). Chromogranin A, which is present in dense secretory granules, is particularly useful in detecting well-differentiated NETs, while synaptophysin, which is associated with synaptic vesicles, has a higher sensitivity and is usually present in both NETs and NECs (1, 19). However, the expression of these markers may vary depending on the degree of tumor differentiation. Poorly differentiated tumors often show weak, focal, or even absent expression of chromogranin A expression due to the lack of secretory granules (1, 12, 19).

NENs are also classified as mixed NENs and non-NENs. These lesions are characterized as neoplasms whose cells express neuroendocrine and non-neuroendocrine features (1, 20). In the gastroenteropancreatic system, by arbitrary convention, each component should represent ≥ 30% of a neoplasm for the neoplasm to be included in the mixed NEN category; the presence of focal (< 30%) neuroendocrine differentiation may be mentioned in the diagnosis (especially when the component is poorly differentiated) but does not affect the diagnostic categorization (1). In these cases, other complementary markers such as CD56, NSE, and chromogranin B can be used to confirm the neuroendocrine nature of the neoplasm (20–22). Thus, although most NENs are positive for chromogranin A and synaptophysin: however, interpretation must always consider the degree of differentiation, histologic features, and biological behavior of the tumor (1).

#### 2.2.1 Pancreatic neuroendocrine neoplasms

Like other NENs, pancreatic NENs (PanNENs) express synaptophysin and usually chromogranin A, and are classified into pancreatic neuroendocrine tumors (PanNETs), and poorly differentiated pancreatic NECs (PanNECs) (23, 24). For pancreatic mixed NEN cases, one neuroendocrine and the other non-neuroendocrine (usually ductal adenocarcinoma or acinar cell carcinoma), each component accounts for at least 30% of the tumor volume (24).

PanNENs are classified according to the degree of differentiation and are graded based on the mitotic rate and Ki-67 proliferation index, similarly to GEP-NENs (**Table 2**). Despite sharing neuroendocrine characteristics, PanNETs and PanNECs are biologically distinct and belong to different genetic categories (23, 24).

**Table 2:** Classification of pancreatic neuroendocrine neoplasms.

Classification/grade	Ki-67 proliferation index	Mitotic count
Well-differentiated PanNENs: pancreatic neuroendocrine tumors (PanNETs)		
G1 PanNET	< 3%	<2
G2 PanNET	2-20%	2-20
G3 PanNET	>20%	>20
Poorly differentiated PanNENs: pancreatic neuroendocrine carcinomas (PanNECs)		
PanNEC (G3)	>20	>20
Small cell type		>20
Large cell type		>20

The Ki-67 proliferation index is based on the evaluation of ≥ 500 cells in areas of higher nuclear labeling (hotspots). The mitotic count is based on the assessment of mitoses in 50 HPF (0.2 mm^2^ each) in areas of higher density and is expressed as the number of mitoses per 10 HPF (2.0 mm^2^). The final grade is determined based on whichever index (Ki-67 or mitotic) places the tumor in the highest-grade category. For the Ki-67 assessment, casual visual estimation (eyeballing) is not recommended; instead, manual counting using printed images is recommended.

PanNETs are well-differentiated neoplasms of low, intermediate, or high-grade, composed of cells with minimal to moderate atypia, with organoid architectural patterns, often without necrosis (24). They express general markers of neuroendocrine differentiation (chromogranin A and synaptophysin, generally in a diffuse and intense form) and hormones (with generally intense expression, although not always diffuse), and may be orthotopic or ectopic concerning the pancreas (23, 24).

PanNECs, on the other hand, are poorly differentiated, high-grade neoplasms composed of highly atypical, small or medium to large cells expressing neuroendocrine markers (such as synaptophysin in a diffuse but weak form and chromogranin A in a focal or weak form), generally without hormone production (23, 24). It is important to note that PanNECs do not express acinar cell markers such as trypsin, chymotrypsin, or carboxyl ester hydrolase, which are typically detected by BCL10 (23, 24).

Finally, mixed NENs represent a conceptual category rather than a specific diagnostic entity (1). Generally, both components are high-grade (G3), but occasionally they may be G1 or G2. Therefore, each component should be graded separately, with special attention to the neuroendocrine component, as its proliferation index (Ki-67) appears to be the main prognostic determinant of mixed NENs (1, 22).

The phenomenon of grade progression in well-differentiated PanNETs has been increasingly recognized and is associated with a poorer prognosis (25). It has been hypothesized that PanNECs may eventually arise from the progression of a well-differentiated PanNET. However, this transformation has not been convincingly demonstrated (1).

### 2.3 Clinical and epidemiological features

The incidence of NENs is approximately seven new cases per 100,000 person-years and has increased steadily over the past two decades (2, 26–28). Both sexes are equally affected, although there is a slight male predominance in tumors of the digestive tract (27, 28). Most patients are between the sixth and eighth decades of life, but younger individuals may be affected, especially in the presence of hereditary syndromes or in ovarian neoplasms due to the association with mature teratomas. NENs are rare in childhood and the neonatal period (29, 30) (**Tables 3**–**5**).

**Table 3:** Clinicopathological features of gastroenteropancreatic neuroendocrine tumors.

Organ	Subtype	Site	Etiopathogenesis	Epidemiology	Clinical features	Pathological features	IHC	Prognosis
Esophagus	NET	Lower esophagus	Unknown	Extremely rare (0.04–1% of all GEP-NENs); sixth to seventh decade of life; no sex bias	Incidental at endoscopy	Classic NE patterns; spotty necrosis rare	Positive: cytokeratin, chromogranin A, synaptophysin, hormones (serotonin, PP, gastrin, enteroglucagon), VMAT2	Good prognosis (few reports)
Stomach	ECL-cell NET (type 1 associated with CAG, type 2 associated with ZES/MEN1, type 3 sporadic), D-cell NET, G-cell NET, EC-cell NET	Corpus/fundus (ECL-cell and EC-cell NETs), antrum (G-cell and D-cell NETs)	Type 1 ECL-cell NET: hypergastrinemia due to CAG; type 2 ECL-cell NET: ZES-MEN1; type 3 ECL-cell NET: unknown MEN1 LOH in sporadic ECL-cell NET; MEN1 mutation in type 2 ECL-cell NET	0.4 cases/100–000 person-years; seventh decade of life; female predominance; type 1 ECL-cell NETs are the most frequent	No specific symptoms; rare ZES due to antral gastrinoma; hypergastrinemia and low BAO and MAO	Classic NE patterns; spotty necrosis rare	Positive: pancytokeratin, chromogranin A, synaptophysin, CDX2, SSTR2 ECL-cell NET: VMAT2+ and ghrelin G-cell NET: gastrin EC-cell NET: serotonin D-cell NET: somatostatin	Largely depends on stage and grade; variable according to type: type I, excellent prognosis; type III, worst; type II, intermediate
Small intestine and ampulla	D-cell NET, G-cell NET, EC-cell NET	Duodenum/ampulla (D-cell NET, G-cell NET), ileum (EC-cell NET)	Unknown; a minority arises in the setting of hereditary cancer predisposition syndrome, e.g. MEN1, NF1 Duodenum and ampulla: MEN1 mutation; small intestine: chromosome 18 deletion; CpG island methylation; CDKN1B mutation; whole-arm copy-number variations	1.2 cases/100–000 person-years; sixth to seventh decade of life; no sex bias	No specific mass-related symptoms; possible transit obstruction and occlusion; vague persisting pain Hormonal syndromes: ZES by gastrinoma; somatostatinoma syndrome; carcinoid syndrome	Classic NE patterns; usually solid islets; spotty necrosis rare; glandular pattern with psammoma bodies in the duodenum (D-cell NETs)	Positive: pancytokeratin, chromogranin A, synaptophysin, CDX2, and SSTR2–5 G-cell NET: gastrin EC-cell NET: VMAT1 and serotonin D-cell NET: somatostatin	Largely depends on stage and grade Ampullary NET: 10-year survival rate of 71% Benign NET of ileum: 5-year survival rates of 70–100% when localized, 35–60% with distant metastases
Colonrectum	L-cell NET, EC-cell NET	Anywhere	Unknown	1.2 cases/100–000 person-years in rectum; 0.2 cases/100–000 person-years in colon; sixth to seventh decade of life; no sex bias	Nonspecific mass-related symptoms	Classic NE patterns; usually solid islets (EC-cell NET); trabeculae/glands (L-cell NET)	Positive: pancytokeratin, chromogranin A, synaptophysin, CDX2, and SSTR2 EC-cell NET: serotonin+ L-cell NET: chromogranin A− and enteroglucagon/PYY+ and PAP	Largely depends on stage and grade; for low-stage G1–G2, the median OS is 30 years (rectum) or 12 years (colon); G3 NET has poor OS (12 months)
Appendix	EC-cell NET, L-cell NET	Corpus to tip	Rare chromosome 18 deletions	0.5–0.6 cases/100–000 person-years; fifth most frequent GEP-NET; third to fourth decade of life, and in children; female prevalence	Nonspecific symptoms; identified incidentally after surgery for appendicitis	Classic NE patterns; usually solid islets (EC-cell NET); trabeculae/glands (L-cell NET)	Positive: pancytokeratin, chromogranin A, synaptophysin, CDX2, and SSTR2 EC-cell NET: serotonin+ L-cell NET: chromogranin A− and enteroglucagon/PYY+	Largely depends on stage and grade; excellent outcome (10-year survival rate of 92%)
Pancreas	NET	Tail and body	MEN1 inactivation; DAXX or ATRX mutation, Germline alterations affecting MEN1, VHL, NF1, GCGR, and MAFA	2–5%, of all pancreatic neoplasms, the estimated incidence of < 1 case per 100–000 person-years, the fourth-seventh decade of life, no sex predilection	Functioning PanNETs present with characteristic hormonal paraneoplastic syndromes, Nonfunctional tumors are encountered incidentally	Classic NE patterns; usually solid islets, glandular patterns with psammoma bodies in somatostatinomas, and amyloid deposition in insulinomas	Positive: pancytokeratin, chromogranin A, synaptophysin	Overall survival rates of 33% at 5 years, 17% at 10 years, and 10% at 20 years
Liver	NET	Anywhere	Unknown; occasional patients have a history of viral hepatitis	Extremely rare; metastatic nature must be excluded; 0.4% of all resected hepatic primaries	Nonspecific mass-related symptoms	Classic NE patterns	Positive: pancytokeratin, chromogranin A, synaptophysin	Long survival when amenable to surgery; 18–47% metastatic disease (G2)
Gallbladder and bile ducts	NET	Gallbladder > bile ducts	Unknown; some associated with VHL and MEN1	Extremely rare (0.21% of all NETs)	Nonspecific mass-related symptoms	Classic NE patterns	Positive: pancytokeratin, chromogranin A, synaptophysin	Limited data; depends on size (larger tumors extend into the liver); 36% OS at 10 years

BAO, basal acid output; CAG, chronic atrophic gastritis; EC, enterochromaffin; ECL, enterochromaffin-like; GEP-NEN, gastroenteropancreatic neuroendocrine neoplasm; GEP-NET, gastroenteropancreatic neuroendocrine tumor; IHC, immunohistochemistry; LOH, loss of heterozygosity; MAO, maximal acid output; MEN1, multiple endocrine neoplasia type 1; n/a, not available; NE, neuroendocrine; NF1, neurofibromatosis type 1; OS, overall survival; VHL, TTF1, thyroid transcription factor 1; von Hippel–Lindau syndrome; ZES, Zollinger–Ellison syndrome.

**Table 4:** Clinicopathological features of gastroenteropancreatic neuroendocrine carcinomas.

Organ	Site	Etiopathogenesis	Epidemiology	Clinical features	Pathological features	IHC	Grading	Prognosis
Small cell NEC								
Esophagus	> 50% of cases in the lower third	Possible risk factors are tobacco smoking, alcohol drinking (no definitive data), Most frequently *TP53* and *RB1* (60% and 54%, respectively); *NOTCH1/3*, *PIK3CA*, and *ATM* mutations more frequently than pulmonary SCNEC	<1% of all digestive NENs; together with LC-NEC, 90% of esophageal NENs; LC-NEC/SC-NEC prevalence variable; male predominance; mean age: 56 years	Nonspecific mass-related symptoms (dysphagia) and weight loss	SC-NEC classic	Positive: AE1/AE3, CAM5.2 dot-like or diffuse, TTF1 in 70%, Variable: chromogranin A, synaptophysin	High-grade by definition, Mitotic rate: > 20 mitoses/2 mm^2^; Ki-67: > 20%	Median OS time range: 8–15 months; older age, upper third location, advanced stage, and no surgery radiotherapy, or chemotherapy are adverse prognostic factors
Stomach	Potentially arise in any part of the stomach, usually in antral and cardiac regions	More frequent in Japanese people; rare gastric primary MCC associated with MCPyV, Most frequently *TP53*; other mutations in *TSHZ3*, *SEMA5A*, *TPH2*, *SDK1*, *PLXNA1*; *RB1* mutations are rare; microsatellite instability in ~10%	20.5% of all digestive NECs; 21% of all gastric NENs; male predominance; mean age: 63 years	Nonspecific mass-related symptoms (dyspepsia) and weight loss	SC-NEC classic	Positive: AE1/AE3, CAM5.2 dot-like or diffuse, TTF1 in 70%, Variable: chromogranin A, synaptophysin, CDX2	High-grade by definition, Mitotic rate: > 20 mitoses/2 mm^2^; Ki-67: > 20%	Poor prognosis; survival time: a few months (no large studies available)
Small intestine and ampulla	Almost exclusively ampullary region	Unknown	Rare	Obstructive effect of mass (mostly obstructive jaundice in ampullary mass)	SC-NEC classic	Positive: AE1/AE3, CAM5.2 dot-like or diffuse, Variable: chromogranin A, synaptophysin	High-grade by definition, Mitotic rate: > 20 mitoses/2 mm^2^; Ki-67: > 20%	Poor prognosis; survival time: a few months (no large studies available)
Colonrectum	Colon and rectum, with similar frequency	Unknown (possibly linked to ADC); Usually mutations in *TP53* and *RB1*; other mutations in *APC*, *KRAS*, *FHIT*, *DCC*, *SMAD4*, *MEN1*, *BRAF*; microsatellite instability in ~10%	Increasing incidence; ~10% of all extrapulmonary small cell carcinomas; slight male predominance; sixth or seventh decade of life	Nonspecific mass-related symptoms, bleeding, or metastasis-related symptoms; the majority of cases are at an advanced metastatic stage at diagnosis	SC-NEC classic	Positive: AE1/AE3, CAM5.2 dot-like or diffuse, CDX2 (usually), Variable: chromogranin A, synaptophysin, TTF1	High-grade by definition, Mitotic rate: > 20 mitoses/2 mm^2^; Ki-67: > 20%	Median OS times for patients with extensive and limited disease: 4.04 months and 21.82 months, respectively; administration of chemotherapy and radiotherapy but not surgery are associated with improved survival
Appendix	No specific localization	Unknown	Very rare (case reports)	Pain, nausea, and vomiting (simulating appendicitis), or nonspecific mass-related symptoms	SC-NEC classic	Positive: AE1/AE3, CAM5.2 dot-like or diffuse, Variable: chromogranin A, synaptophysin, TTF1	High-grade by definition, Mitotic rate: > 20 mitoses/2 mm^2^; Ki-67: > 20%	Poor prognosis; no large studies available
Pancreas	Head	Unknown; mutation in *TP53* and loss of *RB1*	Extremely rare (<2% of pancreatic carcinomas); usually in men (average age 60).	Non-specific symptoms: abdominal pain, weight loss, jaundice (if pancreatic head involvement). Paraneoplastic syndromes are rare.	SC-NEC classic	Positive: AE1/AE3, CAM5.2 dot-like or diffuse, Variable: chromogranin A, synaptophysin, TTF1	High-grade by definition, Mitotic rate: > 20 mitoses/2 mm2; Ki-67: > 20%	Median survival of 6–12 months. High rate of metastases at diagnosis.
Liver	No specific localization	Unknown; occasional patients have a history of viral hepatitis	Very rare; metastatic nature must be excluded in all cases	Nonspecific	SC-NEC classic	Positive: AE1/AE3, CAM5.2 dot-like or diffuse, Variable: chromogranin A, synaptophysin	High-grade by definition, Mitotic rate: > 20 mitoses/2 mm^2^; Ki-67: > 20%	Median survival time: 2 months
Gallbladder and bile ducts	No specific localization	Frequently associated with gallstones; Few data; whole-genome sequencing in a metastatic case showed alterations in *ERBB4*, *HRAS*, *NRG1*, *HMCN1*, *CDH10*, fusions of *NCAM2::SGCZ* and *BTG3::CCDC40*, and microsatellite instability	4% of all malignant gallbladder neoplasms; female predominance; seventh decade of life	Abdominal pain, jaundice, weight loss, ascites, abdominal distension or mass; distant metastases including in the CNS	SC-NEC classic	Positive: AE1/AE3, CAM5.2 dot-like or diffuse, Variable: chromogranin A, synaptophysin	High-grade by definition, Mitotic rate: > 20 mitoses/2 mm^2^; Ki-67: > 20%	Median survival time: 3 months
Large cell NEC								
Esophagus	Mostly in the lower third	Possible risk factors: tobacco smoking, alcohol drinking; specifically LC-NEC related: gastroesophageal reflux disease; Common *TP53* and *RB1* mutations	Rare; as many as half of all oesophageal NECs; M > F; mean age: 70 years	Nonspecific mass-related symptoms (dysphagia) and weight loss	LC-NEC classic; may be associated with a non-NE component (squamous cell carcinoma or ADC)	AE1/AE3, CAM5.2 dot-like or diffuse; synaptophysin (100%), chromogranin A (60%), p63 (40%), TTF1 (40%), CK8/18 (100%), KIT (CD117) (60%), and p16 (60%)	High-grade by definition, Mitotic count > 20 mitoses/2 mm^2^; Ki-67 PI > 20%	Median OS time: 8–15 months
Stomach	Anywhere	Possible risk factors: *Helicobacter pylori*-related atrophic gastritis; Common TP53 mutations; RB1 mutations virtually absent; *TP53*-wildtype cases may have MSI-H; higher frequency of APC mutations than pulmonary NECs, and lower frequency of *KRAS* and *BRAF* mutations than colonic NECs; monoclonal origin in cases with a non-NEC component / MiNEN (sharing altered p53 pathway or MMR defect)	Rare; reported to be more frequent than SC-NEC; M > F; median age: 70 years	Nonspecific mass-related symptoms (dyspepsia) and weight loss	LC-NEC classic; frequently associated with ADC; may be associated with an SC-NEC component	AE1/AE3, CAM5.2 dot-like or diffuse; synaptophysin (90%); chromogranin A (85%); ASH1L (32%); TTF1 (35%)	High-grade by definition, Mitotic count > 20 mitoses/2 mm^2^; Ki-67 PI > 20%	Poor prognosis, overlapping with SC-NEC; 5-year OS rate: 8–66%
Small intestine and ampulla	Almost exclusively in the ampullary region	Unknown; To be defined; may be related to non-NE epithelial neoplasms of the same anatomical site	Rare; reported to be more frequent than SC-NEC; M > F; median age: 70 years	Mostly obstructive jaundice in ampullary mass	LC-NEC classic; may be associated with an adenoma or ADC	Cytokeratins and general NE markers (no systematic study)	High-grade by definition, Mitotic count > 20 mitoses/2 mm^2^; Ki-67 PI > 20%	Median OS time: 11.8 months
Colonrectum	Roughly equal distribution between the right colon and rectosigmoid, rare in the left/descending colon	Possible risk factors: family history of cancer, tobacco smoking, alcohol consumption, and increased body mass index, with the adjusted summary effect estimate of risk (odds ratio) ranging from 0.67 (increased body mass index) to 1.6 (alcohol consumption); Common mutations in *KRAS*, *TP53*, and *APC*; compared with CRC: higher rate of BRAF p.V600E and distinct methylome; EGFR methylated in NECs but not in CRC (different patterns of response and resistance to targeted therapies); possible deregulation of the RB1/p16 pathway and mutations in *FHIT*, *DCC*, *SMAD4*, and *MEN1*; a subset of LC-NECs have MSI-H; monoclonal origin in cases with a non-NEC component / MiNEN	< 1% of all colorectal cancers; no reported sex differences	Nonspecific mass-related symptoms	LC-NEC classic; about half associated with an adenoma and/or ADC, rare cases with a squamous cell carcinoma component	91–100% of cases positive for chromogranin and/or synaptophysin	High-grade by definition, Mitotic count > 20 mitoses/2 mm^2^; Ki-67 PI > 20%	Ominous outcome related to Ki-67 PI: < 55%, median OS: 25.4 months; > 55%, median OS: 5.3 months; LC-NECs with MSI-H may have better OS than non–MSI-H counterparts
Appendix	Not specifically investigated	Unknown	These neoplasms are poorly defined in this location and only anecdotal cases are reported	Not specifically investigated	Not specifically investigated	Not specifically investigated	High-grade by definition, Mitotic count > 20 mitoses/2 mm^2^; Ki-67 PI > 20%	Not specifically investigated
Pancreas	Head	Unknown; Frequent changes in *TP53*, *RB1*, and *KRAS*	Very rare (<1% of pancreatic carcinomas). Average age: 60–70 years. Slight male predominance.	Pain, weight loss, jaundice	LC-NEC classic	Positive: AE1/AE3, CAM5.2 dot-like or diffuse, Variable: chromogranin A, synaptophysin	High-grade by definition Mitotic count > 20 mitoses/2 mm2; Ki-67 PI > 20%	Median survival of 8–14 months. Frequent liver and lymph node metastases at diagnosis.
Liver	Anywhere	Unknown; occasional patients have a history of viral hepatitis (HCV or HBV); To be defined	Extremely rare (0.48% of all hepatic malignancies); metastatic nature must be excluded; almost all occur in association with HCC; combined NEC–intrahepatic cholangiocarcinoma is extremely rare	Nonspecific mass-related symptoms	LC-NEC classic; typically mixed with non-NEC components (HCC)	Synaptophysin+, chromogranin+/−, hepatocyte markers −, albumin (ISH) −, Ki-67 PI > 80%	High-grade by definition, Mitotic count > 20 mitoses/2 mm^2^; Ki-67 PI > 20%	Worse than pure HCC; analysis of a small number of reported cases revealed a 1-year cumulative survival rate of 53%
Gallbladder and bile ducts	Anywhere in the gallbladder (44% in the fundus); in EHBDs, more common in the distal bile duct	Possible risk factors: gallstones, *Clonorchis sinensis* infection; Common *TP53* and *RB1* mutations; other alterations in *ERBB4*, *HRAS*, *NRG1*, *HMCN1*, and *CDH10*, fusions of *NCAM2::SGCZ* and *BTG3::CCDC40*, and microsatellite instability; no *BRAF* mutations	< 1% of all malignant gallbladder and EHBD neoplasms; F>M; average age in sixth decade of life	Abdominal pain, jaundice, weight loss, ascites, abdominal distension or mass	LC-NEC classic; one-third of cases mixed with ADC or SC-NEC	AE1/AE3, CAM5.2 dot-like or diffuse; synaptophysin (100%), chromogranin A (53%)	High-grade by definition, Mitotic count > 20 mitoses/2 mm^2^; Ki-67 PI > 20%	Median survival time: < 1 year; 5-year OS rate: 20%; 10-year OS rate: 0%

ADC, adenocarcinoma; CRC, colorectal carcinoma; EC, enterochromaffin; ECL, enterochromaffin-like; EHBD, extrahepatic bile duct; F, female; HCC, hepatocellular carcinoma; GEP-NEN, gastroenteropancreatic neuroendocrine neoplasm; GEP-NET, gastroenteropancreatic neuroendocrine tumor; IHC, immunohistochemistry; ISH, *in situ* hybridization; M, male; MCC, Merkel cell carcinoma; MCPyV, Merkel cell polyomavirus; MMR, mismatch repair; MSI-H, high level of microsatellite instability; NEC, neuroendocrine carcinoma; OS, overall survival; PI, proliferation index; LC-NEC, large cell neuroendocrine carcinoma; SC-NEC, small cell neuroendocrine carcinoma; TTF1, thyroid transcription factor 1.

**Table 5:** Clinicopathological features of gastroenteropancreatic mixed neuroendocrine and non-neuroendocrine neoplasms.

Organ	Subtype	Site	Etiopathogenesis	Epidemiology	Clinical features	Pathological features	IHC	Grading	Prognosis
Esophagus	ADC-NEC, SCC-NEC, ADC-NET	ADC-NEC: distal esophagus, SCC-NEC: any location, ADC-NET: distal esophagus	Probably the same as that of ADC and SCC, *TP53* and *RB1* mutations, *RB1* deletion or LOH, and amplification of *PIK3CA*, *PTEN*, *KRAS*, *SOX2*, *DVL3*, and *TP63*	0.2–4% of all esophageal malignancies; 6–16% of all digestive MiNENs, mean age: 67 years	Same as those of ADC and SCC	Conventional features of each component	SCC: CK5/6, p63, p40, ADC: CK7, CK19; NEC: synaptophysin, chromogranin A, TTF1; NET: synaptophysin, chromogranin A	ADC and SCC: graded as pure forms; NEC: high-grade by definition, NET: graded (G1, G2, or G3) according to proliferative index	Median survival is about 6 months depending on the tumor stage
Stomach	ADC-NEC, ADC-NET	Any location	ADC-NEC: similar to that of pure ADC, ADC-NET: unknown	0.16–1.48% of all gastric malignancies; 6–20% of all digestive MiNENs, mean age: 69 years	Same as those of ADC, paraneoplastic symptoms are uncommon	Conventional features of each component or signet-ring cell in ADC	ADC: CK7, CK19; NEC: synaptophysin, chromogranin A, TTF1+/−; NET: synaptophysin, chromogranin A	ADC: graded as pure forms; NEC: high-grade by definition, NET: graded (G1, G2, or G3) according to proliferative index	ADC-NEC: median survival time: 27 months; 5-year survival rate: 8–11% with advanced-stage disease even after surgical resection; ADC-NET: better prognosis than ADC-NEC
Small intestine and ampulla	ADC-NEC, ADC-NET	Duodenum and ampullar region	ADC-NEC: similar to that of pure ADC, ADC-NET: similar to that of pure ADC (*KRAS* and *TP53* mutation demonstrated in 1 case)	5.6% of all digestive MiNENs	Same as those of ADC, paraneoplastic hormonal symptoms are uncommon	Conventional features of each component or signet-ring cell in ADC	ADC: CK7, CK19; NEC: synaptophysin, chromogranin A, TTF1+/−; NET: synaptophysin, chromogranin A	ADC: graded as pure forms; NEC: high-grade by definition, NET: graded (G1, G2, or G3) according to proliferative index	ADC-NEC: mean survival time: 61 months; ADC-NET: better prognosis than ADC-NEC
Colonrectum	ADC-NEC, ADC-NET	Any location	ADC-NEC: similar to that of pure ADC, ADC-NET: similar to that of pure ADC (*KRAS*, *AKT1*, *APC*, *PIK3CA*, *SMAD4*, *RB1*, and *TP53* mutations demonstrated)	30% of all digestive MiNENs, median age at diagnosis: 65 years	Same as those of ADC, paraneoplastic hormonal symptoms are uncommon	Conventional features of each component or signet-ring cell in ADC	ADC: CK20, CDX2; NEC: synaptophysin, chromogranin A, TTF1+/−; NET: synaptophysin, chromogranin A	ADC: graded as pure forms; NEC: high-grade by definition, NET: graded (G1, G2, or G3) according to proliferative index	Median OS time of 29.6 or 9.6 months (localized and advanced disease respectively)
Appendix	ADC-NEC, ADC-NET	Any location	ADC-NEC: similar to that of pure ADC	1% of all appendiceal epithelial malignancies, Median age: 57 years	Same as those of ADC, appendicitis	Conventional features of each component or signet-ring cell in ADC	ADC: CK20, CDX2; NEC: synaptophysin, chromogranin A, TTF1; NET: synaptophysin, chromogranin A	ADC: graded as pure forms; NEC: high-grade by definition, NET: graded (G1, G2, or G3) according to proliferative index	5-year OS rate: 57.4%, 5-year DSS rate: 36.4%; worse than NET, NEC, and goblet cell ADC
Pancreas	ACC-NEC; ACC-NET; DADC-NEC; DADC-NET	Any location	ACC-NEC: similar to that of pure ACC; DADC-NEC: similar to that of pure ADC;	0.2–2% of all pancreatic malignancies; median age: 61–67 years	Same as those of ACC and DADC	Conventional features of each component	ACC: trypsin, chymotrypsin, BCL10 DADC: CEA, EMA (MUC1), and/or MUC2; NEC: synaptophysin, chromogranin A, TTF1, INSM1; NET: synaptophysin, chromogranin A	ACC and DC: graded as pure forms; NEC: high-grade by definition, NET: graded (G1, G2, or G3) according to proliferative index	ACC: 5-year OS rate: 30–50%; DADC; Patients rarely survive > 3 years. The 2-year and 5-year survival rates are 25% and 0%, respectively. Mixed DADC–NECs have a slightly longer median survival time than pure NECs (20 months vs 12 months) Mixed DADC–NETs seem to show better survival than mixed DADC-NET
Liver	HCC-NEC, HCC-NET, CHC-NEC	Any location	Unknown	Extremely rare	Same as those of liver carcinomas	Conventional features of each component	HCC: HepPar1, arginase, GPC3, GS; CHC: CK7; NEC: synaptophysin, chromogranin A, TTF1 +/−	HCC and CHC: graded as pure forms; NEC: high-grade by definition, NET: graded (G1, G2, or G3) according to proliferative index	No specific information on OS, but these are aggressive neoplasms
Gallbladder and bile ducts	ADC-NEC, ADC-ICPN-NEC	Any location	ADC-NEC: similar to that of pure ADC (*TP53* mutation demonstrated)	10% of gallbladder carcinomas; 4% of extrahepatic cholangiocarcinomas; 2.4% of all digestive MiNENs, median age at diagnosis: 65 years (range: 34–85 years); M:F ratio: 1:3.3	Same as those of ADC, paraneoplastic hormonal symptoms are uncommon	Conventional features of each component or signet-ring cell in ADC	ADC: EMA, CK7; ICPN: EMA, MUC5A, CK7; NEC: synaptophysin, chromogranin A, TTF1 +/−	ADC and SCC: graded as pure forms; NEC: high-grade by definition, NET: graded (G1, G2, or G3) according to proliferative index	Median OS time of 8.6 or 4.4 months (localized and advanced disease respectively)

ACC, acinar cell carcinoma; ADC, adenocarcinoma; DADC, ductal adenocarcinoma; DSS, disease-specific survival; CHC, cholangiocarcinoma; HCC, hepatocellular carcinoma; ICPN, intracholecystic papillary neoplasm; IHC, immunohistochemistry; LOH, loss of heterozygosity; NEC, neuroendocrine carcinoma; NET, neuroendocrine tumor, OS, overall survival; SCC, squamous cell carcinoma; TTF1, thyroid transcription factor 1.

Most tumors are non-functional and therefore do not show signs or symptoms associated with active hormone secretion (1). Since they may remain asymptomatic for long periods, they are usually diagnosed at more advanced stages due to the absence of early clinical signs related to hormone secretion (31). In these cases, patients usually present with non-specific symptoms related to mass effect or incidental findings on examinations performed for other reasons (1).

In functional NENs, excessive production of their substances is associated with specific clinical presentations and the development of syndromic patterns (1). It is estimated that less than one-third of NENs are associated with hormonal syndromes, and they are categorized according to the predominant hormone secreted, such as carcinoid syndrome (due to serotonin secretion), Zollinger-Ellison syndrome (related to gastrinomas), recurrent hypoglycemia (in insulinomas), hyperglycemia and migratory necrolytic erythema (in glucagonomas), Verner-Morrison syndrome (in VIPomas), and manifestations such as diabetes, cholelithiasis, and steatorrhea (in somatostatinomas), among others (24, 32–34).

## 3 Metabolism in cancer

### 3.1 Carbohydrate metabolism

One of the most well-established hallmarks of cancer is metabolic reprogramming, particularly involving carbohydrate metabolism. The Warburg effect, first described by Otto Warburg, denotes the preferential use of aerobic glycolysis over oxidative phosphorylation, even under normoxic conditions. Although this pathway generates less ATP, it enables the continuous supply of intermediates necessary for rapid proliferation (35–37).

This metabolic shift is orchestrated by oncogenes such as myelocytomatosis oncogene (*MYC)*, rat sarcoma virus oncogene (*RAS)*, and protein kinase B (*AKT)*, and transcriptional regulators like hypoxia-inducible factor 1 (HIF-1), while tumor protein p53 (TP53) downregulation further enhances glycolytic flux (35).

Consequently, tumor cells upregulate glucose transporters (GLUTs), particularly GLUT1, which is commonly overexpressed in several malignancies and represents a potential therapeutic target (38–40). Additionally, transporters such as SLC50A1 (SWEET1) have emerged as promising diagnostic biomarkers due to their strong association with tumor grade and prognosis (41).

Key glycolytic enzymes, including hexokinase 2 (HK2), pyruvate kinase M2 (PKM2), and lactate dehydrogenase A (LDHA), sustain the glycolytic phenotype. These enzymes promote lactate accumulation and acidification of the tumor microenvironment. This contributes to invasion and treatment resistance (42, 43). They are also regulated at the post-transcriptional level by microRNAs, such as miR-145, which modulate glycolysis and mTOR signaling (44–46).

Beyond glucose, tumor cells exhibit remarkable metabolic flexibility, utilizing other carbohydrates such as fructose, galactose, and mannose (47–49). The uptake of fructose via GLUT5 (SLC2A5) and the production of fructose endogenously through the polyol pathway (mediated by *AKR1B1* and *SORD*) sustain glycolysis under hypoxic and acidic conditions. These processes have also been linked to epithelial-mesenchymal transition (EMT) and poor clinical outcomes (49–52). Similarly, alterations in galactose metabolism via the Leloir pathway affect glycosylation and cell signaling, thereby influencing tumor growth and therapeutic response (53–55). Conversely, mannose metabolism can have anti-tumor effects. Accumulation of mannose-6-phosphate inhibits glycolysis and enhances oxidative stress, making cancer cells more susceptible to chemotherapy (56–61).

### 3.2 Lipid metabolism

Although glucose and glutamine metabolism have been extensively studied, the crucial and multifaceted role of lipid metabolism in cancer progression has recently received significant attention (62, 63). Lipids, including fatty acids, cholesterol, and phospholipids, are essential for membrane structure, cellular signaling, energy storage, and the regulation of proliferation and survival. Alterations in lipid composition affect membrane dynamics and signaling, thereby influencing processes such as cell growth, apoptosis, motility, and metastasis (64, 65).

Enhanced lipid synthesis and fatty acid β-oxidation support tumor proliferation by supplying membrane components, generating energy, and producing lipid-derived signaling molecules. In particular, fatty acids and phospholipids contribute to tumor development, migration, and invasion, and their metabolic pathways represent potential therapeutic targets (66). A pivotal mediator of this enhanced lipogenesis is fatty acid synthase (FASN), an enzyme responsible for catalyzing the synthesis of long-chain fatty acids, whose overexpression has been associated with increased tumor proliferation and poor clinical outcomes (67).

Reprogramming cholesterol metabolism promotes aggressive tumor phenotypes, including therapy resistance and metastatic potential. This occurs largely through modulation of lipid rafts and oncogenic signaling (68–70). These processes are tightly controlled by oncogenic pathways, such as PI3K/Akt, Wnt/β-catenin, and STAT3, as well as by non-coding RNAs that regulate key enzymes, including SREBP, FASN, and PPARs (66). Overall, lipid metabolic reprogramming is a hallmark of oncogenesis, linking metabolism to tumor behavior and providing a framework for identifying metabolic biomarkers with diagnostic and prognostic potential in cancer.

### 3.3 Amino acid metabolism

Amino acids are key regulators of metabolic and signaling networks that sustain tumor growth and proliferation. Metabolic reprogramming is a hallmark of cancer that reflects tumor-intrinsic properties and microenvironmental influences. It encompasses alterations in transporter activity, biosynthesis, and catabolism (71).

Glutamine plays a central role among them as an anaplerotic substrate that fuels the TCA cycle through glutaminolysis. This process generates intermediates such as α-ketoglutarate (α-KG) and oxaloacetate (OAA), which are essential for mitochondrial ATP production (71, 72). Under hypoxic or glucose-limited conditions, glutamine metabolism adapts through reductive carboxylation to enable citrate production and sustain biosynthetic processes critical for tumor survival.

Glutamine uptake and utilization are highly heterogeneous across tumor types, modulated by factors such as oncogenic signaling and hypoxia (73, 74). Although glutaminase (GLS) is a pivotal enzyme in glutamine catabolism, its inhibition alone is often insufficient due to metabolic plasticity and compensatory pathways involving other amino acids (75–77). Broader targeting strategies that disrupt both anaplerotic and nitrogen metabolism have demonstrated improved antitumor efficacy (78, 79).

Furthermore, perturbations in glutamine availability can reshape the tumor immune microenvironment by reducing PD-L1 expression, reprogramming macrophage polarization, and enhancing immunotherapy response (80, 81). Glutamine deprivation also increases tumor reliance on other amino acids, such as aspartate, asparagine, and branched-chain amino acids (BCAAs: leucine, isoleucine, and valine), which support biosynthesis and redox balance (82, 83). Together, these adaptive mechanisms demonstrate the importance of amino acid metabolism in cancer biology. Understanding how tumors rewire these pathways provides a foundation for identifying metabolic biomarkers with diagnostic and prognostic potential and for designing therapies that exploit metabolic vulnerabilities.

### 3.4 Nucleotide metabolism

Nucleotide metabolism is fundamental to nucleic acid synthesis, energy homeostasis, and intracellular signaling (84). The balance between synthesis and recycling is maintained through two main pathways: the *de novo* pathway, which produces nucleotides from metabolic precursors such as glucose and glutamine, and the salvage pathway, which recycles nitrogenous bases and nucleosides (84, 85).

In normal cells, nucleotide biosynthesis is tightly coupled to proliferation signals. Transcription factors such as *MYC* and *Rb/E2F* regulate key enzymes during the cell cycle. However, in cancer, these pathways are extensively reprogrammed to sustain uncontrolled proliferation and genomic instability. Different tumor subtypes have specific dependencies, such as a preference for *de novo* synthesis or the salvage pathway, reflecting metabolic plasticity and adaptive survival strategies (85, 86).

This metabolic flexibility supports not only DNA and RNA synthesis, but also redox regulation and signaling. Consequently, enzymes within nucleotide biosynthetic pathways have become important therapeutic targets. Classic antimetabolites and newer, subtype-specific inhibitors demonstrate how disrupting nucleotide metabolism can hinder tumor growth and expose metabolic vulnerabilities (86, 87).

Together, these insights reinforce the concept that metabolic reprogramming, spanning carbohydrate, lipid, amino acid, and nucleotide metabolism, is integral to tumor progression. This framework provides a foundation for understanding how metabolic biomarkers can inform diagnosis, prognosis, and treatment response, particularly in NENs.

## 4 Methods

This review was conducted as a narrative synthesis of the current literature on the role of metabolism in GEP-NENs. Although not designed as a systematic review, the selection and evaluation of the literature followed a structured and transparent approach to ensure comprehensiveness and relevance.

A targeted search was performed in the PubMed/MEDLINE database from its inception to May 2025 using combinations of controlled vocabulary (MeSH terms) and free-text keywords related to metabolism and GEP-NENs. The main search strategy included terms such as “metabolism”, “metabolic”, “neuroendocrine tumor”, and “neuroendocrine neoplasm”. Additional relevant studies were identified through manual screening of reference lists from retrieved articles and key reviews. No language restrictions were applied.

Studies were considered for inclusion if they provided original data on metabolic alterations associated with the diagnosis, prognostic stratification, or therapeutic response of GEP-NENs. Both clinical and preclinical investigations were included when they offered mechanistic insights into metabolic alterations underlying tumor behavior.

All titles and abstracts were screened for relevance, followed by a full-text evaluation of eligible papers. Given the heterogeneity of study designs and analytical platforms, a qualitative synthesis was prioritized over a quantitative meta-analysis. The findings are therefore presented descriptively, emphasizing emerging patterns, recurrent metabolic pathways, and clinical implications.

## 5 Neuroendocrine neoplasms and metabolic markers

### 5.1 Esophagus

No studies specifically addressing metabolic biomarkers in esophageal neuroendocrine tumors NETs were identified. The available literature primarily focuses on immunohistochemical, genetic, and prognostic aspects of these tumors, with no direct emphasis on pathways or proteins related to cellular metabolism. For instance, one study article evaluated the expression of p16 and RB proteins in esophageal NEC, demonstrating frequent alterations in the p16–RB pathway with prognostic implications, but without relevance to cellular metabolism (88).

### 5.2 Stomach

The ALDH enzyme family plays a critical role in oxidizing both endogenous and exogenous aldehydes. Among its members, ALDH1A1 is primarily responsible for metabolizing acetaldehyde and has been linked to alcohol sensitivity, dependence, and osteosarcoma. In the context of NEMs, immunohistochemical analysis of gastric tumors revealed that 52.2% exhibited positive cytoplasmic staining for ALDH1A1, with 20.9% demonstrating strong positivity. Notably, strong ALDH1A1 expression was significantly associated with adverse clinicopathological features, including lymph node involvement, lymphovascular invasion, and a higher Ki-67 index, and correlated with poorer overall survival. Furthermore, multivariate analysis confirmed that high ALDH1A1 expression, along with lymph node metastasis and lymphovascular invasion, served as independent predictors of decreased overall survival (89).

**Figure 1:**
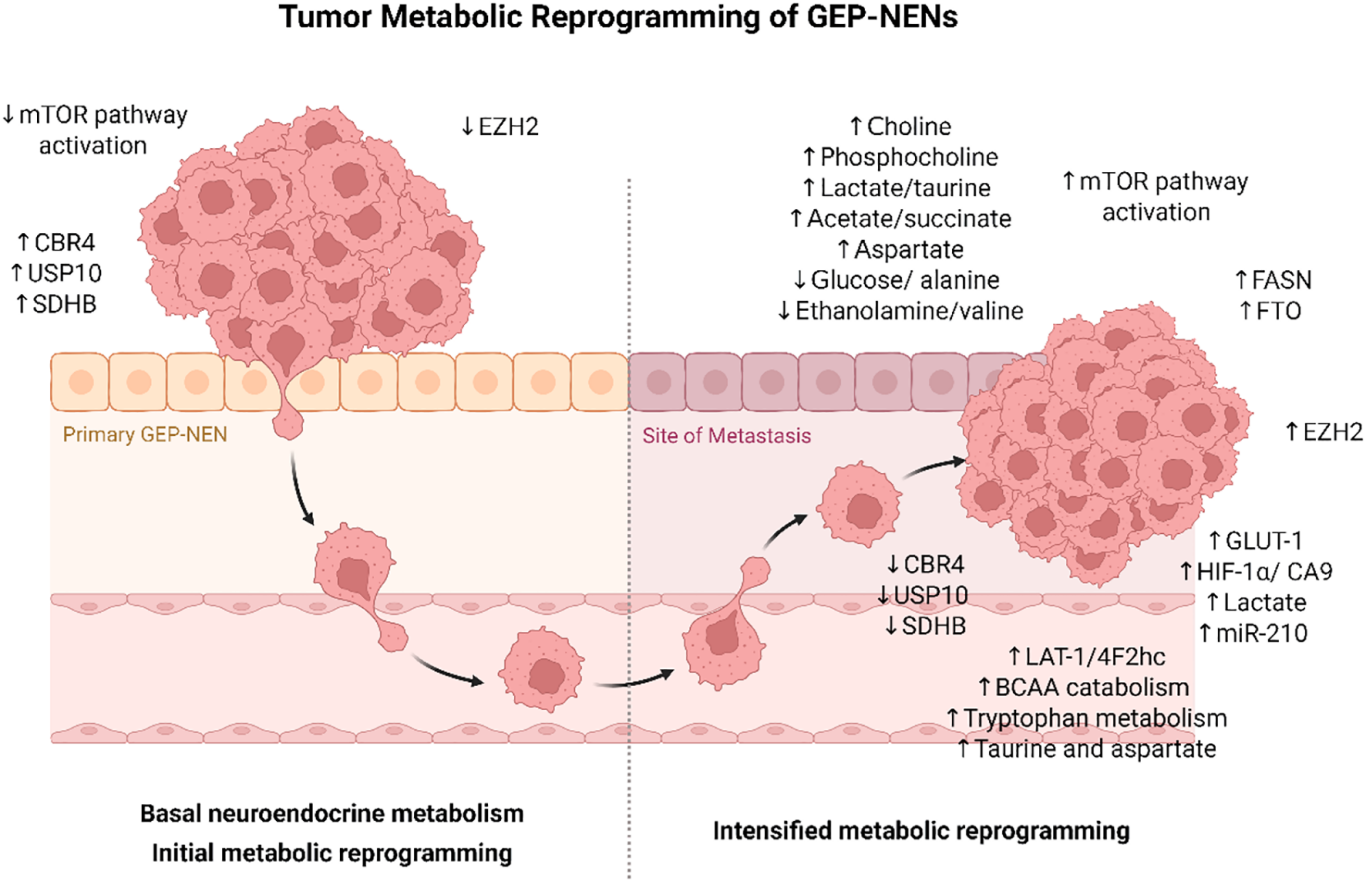
Metabolic reprogramming during progression of gastroenteropancreatic neuroendocrine neoplasms (GEP-NENs). Initial phases of tumor progression are characterized by reduced activation of *mTOR/PI3K/AKT* and *EZH2* signaling, with increased expression of CBR4, USP10, and SDHB. As tumors progress, a shift from basal neuroendocrine metabolism to intensified metabolic reprogramming occurs, marked by elevated levels of choline, phosphocholine, lactate, taurine, acetate, and succinate, along with suppression of aspartate, glucose/alanine, and ethanolamine/valine. Metastatic lesions show upregulation of GLUT-1, HIF-1α, CA9, miR-210, FASN, FTO, and EZH2, along with enhanced BCAA and tryptophan metabolism and increased LAT-1/4F2hc expression. These changes collectively support the metabolic plasticity required for tumor dissemination and adaptation to the metastatic niche.

**Figure 2:**
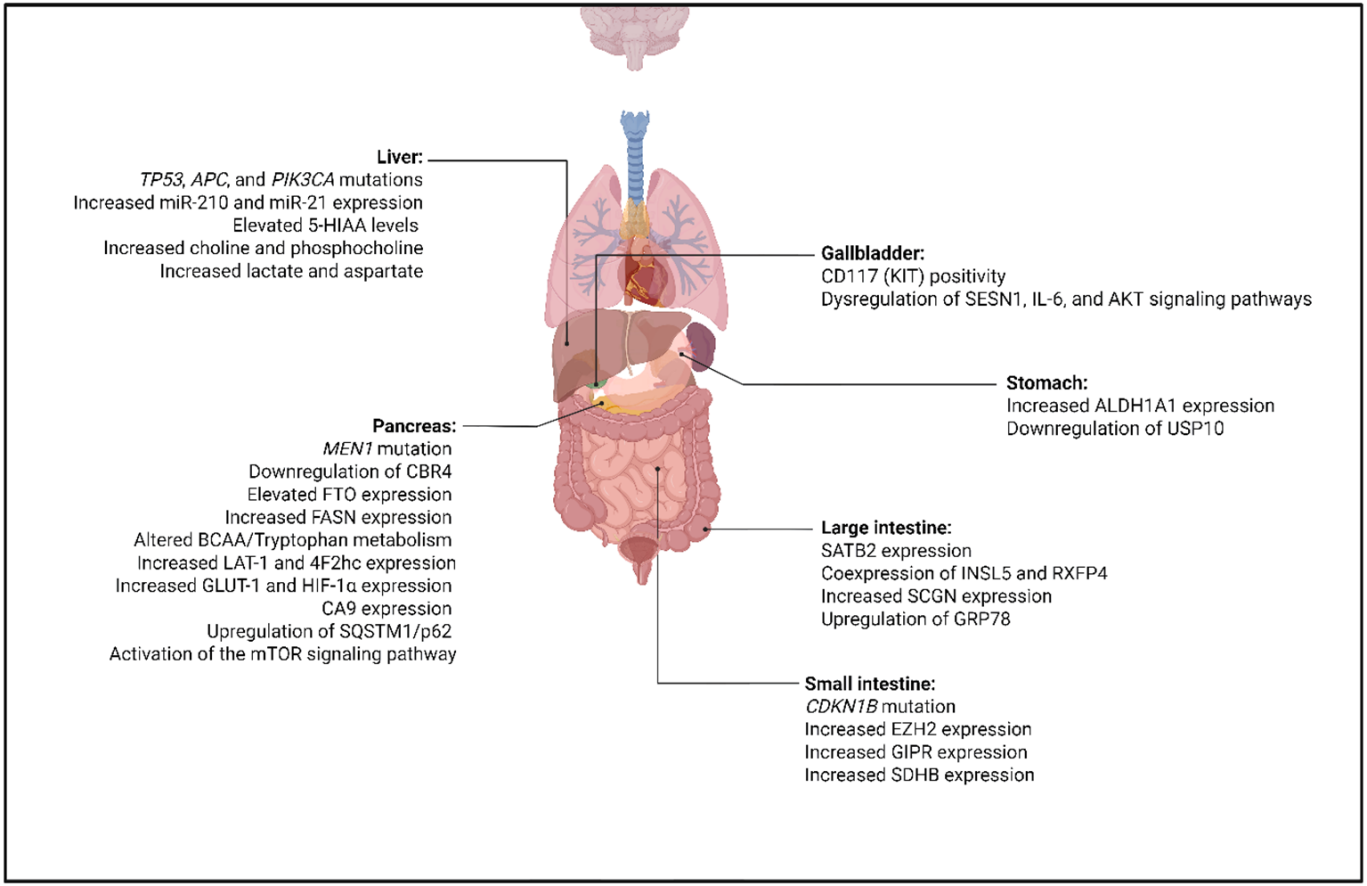
Site-specific molecular and metabolic alterations in gastroenteropancreatic neuroendocrine neoplasms (GEP-NENs). In the liver, metastatic lesions frequently harbor TP53, APC, and PIK3CA mutations, with elevated miR-210 and miR-21 expression and increased levels of 5-HIAA, choline, phosphocholine, lactate, and aspartate. In the pancreas, MEN1 mutations are associated with downregulation of CBR4 and upregulation of FTO, FASN, LAT-1/4F2hc, GLUT-1, HIF-1α, CA9, and SQSTM1/p62, along with altered BCAA and tryptophan metabolism and activation of mTOR signaling. Gallbladder NENs show CD117 (KIT) positivity and dysregulation of SESN1, IL-6, and AKT pathways. Stomach NENs show increased ALDH1A1 expression and decreased USP10 expression. In the colon, SATB2, INSL5, and RXFP4 are expressed with upregulation of SCGN and GRP78. Small intestinal tumors are associated with CDKN1B mutations, increased EZH2, GIPR, and SDHB expression.

Complementing these findings, the role of ubiquitin-specific peptidase 10 (*USP10*), a member of the ubiquitin-specific protease family involved in stress responses, tumor growth, and cellular metabolism, has been investigated in gastric cancer (GC). Expression studies demonstrated that USP10 levels were significantly lower in GC tissues and cell lines compared to non-cancerous gastric mucosa and an immortalized gastric epithelial cell line. Moreover, reduced USP10 expression was inversely correlated with deeper gastric wall invasion, nodal metastasis, and advanced TNM stage, while showing a positive association with E-cadherin expression. Kaplan-Meier analysis indicated that negative USP10 expression was linked to poorer prognosis, and multivariate analysis identified USP10 as an independent prognostic factor for overall survival in GC patients (90) (**Figures 1**, **2**).

### 5.3 Small intestine and ampulla

Growing evidence indicates that epigenetic and metabolic changes in cancer cells are closely linked. EZH2, a key epigenetic regulator, suppresses gene transcription through H3K27me3 trimethylation and exhibits high enzymatic activity in cancer cells. It promotes glucose metabolism and aerobic glycolysis, thereby supporting tumor progression through the Warburg effect (91). Small bowel NETs, although genetically stable, display significant epigenetic dysregulation. Unlike normal enterochromaffin cells, small bowel NETs and their metastases exhibit high EZH2 expression. Silencing EZH2 in CNDT2.5 cells reduced proliferation and induced apoptosis, while EZH2 knockout in a xenograft model inhibited tumor growth. Furthermore, EZH2 inhibitors such as CPI-1205 and GSK126 reduced viability, migration, and proliferation in small intestine NET (SI-NET) cell lines. Metformin has also been shown to suppress EZH2 expression and inhibit proliferation in CNDT2.5 and GOT1 cells (92).

GIPR, a key regulator of postprandial metabolism, is involved in glucose metabolism and insulin sensitivity. Gene expression analysis in gastric and duodenal NETs, as well as in small bowel NETs and PanNETs, revealed significant overexpression of multiple receptors; however, GIPR exhibited the greatest overexpression relative to normal tissue. This suggests that GIPR may offer an improved signal-to-noise ratio for imaging and represents a novel therapeutic target (93).

Cyclin-dependent kinases (CDKs) regulate cell cycle progression, and their deregulation has been associated with various diseases, including cancer. In SI-NETs, 8.5% of patients exhibited pathogenic CDKN1B mutations, including insertions, deletions, nonsense variants, and stop-loss variants. These mutations displayed inter- and intratumor heterogeneity but did not correlate with p27 protein expression or clinical characteristics. CDKN1B is considered a potential haploinsufficient tumor suppressor gene in SI-NETs (94).

Finally, SDHB, a mitochondrial complex II subunit, was detected in all ileal tumor cells. Expression levels were significantly higher in primary specimens compared to metastatic ones. Notably, loss of SDHB expression in metastatic tumors correlated with longer overall survival, suggesting that SDHB, combined with Ki-67%, may serve as a prognostic marker in metastatic SI-NETs (95).

### 5.4 Colorectum and appendix

SATB2, a nuclear matrix-associated protein that regulates gene expression, is normally expressed in the lower gastrointestinal tract. The immunohistochemical analysis identified SATB2 as a sensitive and specific marker for rectal and appendiceal well-differentiated NETs, showing strong expression in 100% of rectal and appendiceal tumors. These findings suggest that SATB2 may serve as a potential diagnostic tool for differentiating primary sites of gastrointestinal NETs (96).

In the context of NEC metabolic biomarkers, INSL5 and its receptor RXFP4 have been identified in colorectal tissues and may play a role in rectal NETs. All examined rectal NETs co-expressed INSL5 and RXFP4, suggesting that INSL5-RXFP4 signaling could be involved in the biology of colorectal NETs (97).

Glucose-regulated proteins (GRPs), cellular proteins responsive to glucose deprivation, play essential roles in protein folding, assembly, and homeostasis. Proteomic analysis of colorectal carcinoma revealed significant alterations in protein expression, with secretagogin (SCGN) downregulated and GRP78 upregulated in tumor tissues. Although GRP78 exhibited increased protein levels, mRNA expression did not differ significantly between tumor samples and normal mucosa. Immunohistochemical analysis demonstrated strong SCGN expression in 98% of neuroendocrine tumors, highlighting its potential as a diagnostic marker (98).

### 5.5 Pancreas

Several studies have highlighted the role of glucose transporters, particularly GLUT-1, as metabolic biomarkers in PanNETs. One study (99) demonstrated significantly increased expression of GLUT-1 and hypoxia-inducible factor 1-alpha (HIF-1α) in grade 2 PanNETs, NECs, and mixed tumors. These markers were correlated with vascular invasion, lymph node metastasis, high Ki-67 index, and shorter disease-free survival. Furthermore, the association between GLUT-1 and HIF-1α (P = 0.025) suggests HIF-1α-driven GLUT-1 upregulation under hypoxic conditions, facilitating glucose uptake and metabolic reprogramming. By contrast, GLUT-2 showed no prognostic relevance in pancreatic tumors, further supporting the specificity of GLUT-1 as a biomarker in PanNETs.

A comprehensive study of GEP-NETs, including PanNETs, demonstrated that GLUT-1 is overexpressed in aggressive tumors and correlates with metastatic disease and reduced VHL (von Hippel–Lindau) expression, reinforcing the activation of HIF-related pathways under hypoxic conditions (100). These findings highlight the potential of GLUT-1 not only as a diagnostic marker but also as a therapeutic target, particularly in high-grade tumors. In addition to glucose metabolism, amino acid metabolism plays a critical role in supporting PanNET growth. The L-type amino acid transporter 1 (LAT-1) and 4F2hc amino acid transporters exhibit distinct expression patterns linked to tumor aggressiveness (100). Specifically, LAT-1 overexpression was associated with metastatic behavior, low pVHL levels, and high GLUT-1 expression, suggesting coordinated metabolic adaptation to hypoxia. Meanwhile, 4F2hc was more frequently expressed in NET-G2/G3 tumors with vascular invasion and elevated Ki-67, serving as both a diagnostic and prognostic marker. Notably, only 4F2hc was proposed as a potential predictor of mTOR inhibitor response, despite showing no significant impact on overall survival (101).

Additionally, GLUT-1 expression is relevant in functional imaging, such as FDG-PET, which is more frequently positive in aggressive PanNETs. A complementary dual-imaging phenotype has been described in PanNETs, reflecting distinct metabolic and molecular profiles. SSTR-PET (e.g., ^68^Ga-DOTATATE) positivity is characteristic of well-differentiated tumors with preserved neuroendocrine features and predominant somatostatin receptor expression. In contrast, FDG-PET positivity correlates with higher proliferative activity, dedifferentiation, and activation of glycolytic pathways (102, 103). The upregulation of glycolytic mediators, such as GLUT-1, in PanNETs has repeatedly been correlated with malignant potential and is often linked to increased HIF-1α expression in hypoxic tumor areas (99, 102). Mechanistically, mTOR signaling can induce HIF activity and thereby promote glycolytic gene expression in pancreatic cells, providing a biological link between growth-promoting pathways and metabolic reprogramming (104). Taken together, these observations suggest that the shift from an SSTR-dominant to an FDG-dominant phenotype reflects metabolic reprogramming during PanNET progression. This integration of imaging, molecular, and metabolic features provides a continuum of tumor aggressiveness.

In addition to glucose metabolism, FASN also emerges as a central metabolic biomarker, commonly overexpressed in PanNETs and associated with metastasis, advanced stages, and poor overall survival (105). FASN inhibition with orlistat-induced ferroptosis, evidenced by decreased xCT and GPX4 expression. Moreover, recent studies demonstrated that FABP5 and the FTO demethylase stabilize FASN, enhancing lipid accumulation and activating key oncogenic pathways such as WNT/β-catenin and PI3K/AKT/mTOR inhibition of FABP5 or FTO decreased tumor cell proliferation and migration, and the combination of orlistat with everolimus enhanced antitumor effects. These findings highlight FASN as both a prognostic marker and a potential therapeutic target (106, 107).

The *MEN1* gene, frequently mutated in PanNETs, also modulates lipid metabolism. It inhibits the mTOR–SCD1 axis, promoting ferroptosis via polyunsaturated fatty acid (PUFA) peroxidation (108). MEN1 overexpression reduces SCD1 levels, sensitizing cells to ferroptosis. The effect is partially reversed by oleic acid, a product of SCD1. Combined treatment with Everolimus and ferroptosis inducers (e.g., RSL3) demonstrated enhanced efficacy in pNET cells with high MEN1 expression.

Additionally, beyond its effect on FASN, FTO also regulates APOE expression in PanNETs, promoting the accumulation of lipids, cholesterol, and triglycerides (107). Inhibition of FTO with FB23 significantly reduced tumor progression, particularly when combined with Everolimus. Additionally, the oxysterol 24S-hydroxycholesterol (24S-HC), synthesized by Cyp46a1, was linked to HIF-1α-mediated angiogenesis. Although this mechanism has been described beyond pancreatic neuroendocrine tumors, inhibition of Cyp46a1 with zaragozic acid or overexpression of SULT2B1b reduced angiogenesis, indicating a potential role of cholesterol metabolism in PanNETs and other GEP-NENs (109).

Carbonyl reductase 4 (CBR4), which negatively regulates FASN, is frequently downregulated in PanNETs as a result of hypoxia-induced promoter methylation. Its low expression was associated with poor prognosis and resistance to Everolimus (110). Overexpression of CBR4 led to FASN degradation, mTOR inhibition, and increased Everolimus sensitivity, supporting its role as a predictive biomarker.

A metabolomic study specifically focusing on metastatic PanNETs (111) identified an enrichment of branched-chain amino acid (BCAA) degradation pathways (involving BCKDHA and BCKDHB) and tryptophan metabolism (via TDO2, DAO, DPYS). The metabolic reprogramming appeared to support energy production through the TCA cycle, contributing to tumor growth and dissemination in PanNETs.

Hypoxia is a critical feature of the PanNET tumor microenvironment, influencing the expression of markers such as carbonic anhydrase 9 (CA9), which is induced by HIF-1α. CA9, a pH-regulating enzyme induced by hypoxia, was expressed in large PanNETs and those associated with von Hippel-Lindau (VHL) syndrome, while absent in microadenomas or small tumors (112). Its presence was associated with aggressive behavior and an unfavorable prognosis.

In parallel, the mTOR signaling pathway is a central metabolic regulator that is frequently activated in PanNETs (113). Markers such as p-mTOR and p-p70S6K have been associated with better response to Everolimus, while p-4EBP1 expression correlated with poor survival. In addition, SQSTM1/p62, involved in autophagy and mTOR signaling, was overexpressed in PanNETs and associated with recurrence and worse outcomes (114). Its inhibition reduced mTOR phosphorylation and tumor proliferation.

Although not a classical metabolic biomarker, insulin-like growth factor 1 (IGF-1) modulates the PI3K/AKT/mTOR pathway and chromogranin A secretion via R-type Ca²^+^ channels (CaV2.3). This mechanism has been observed in neuroendocrine cell lines, but further validation in human PanNETs is needed (115).

### 5.6 Liver

Primary hepatic NENs are rare entities, and their molecular landscape remains poorly understood. TP53 mutations have been identified in both NETs and NECs, reflecting genomic instability and contributing to tumor metabolic plasticity (116). TP53 dysfunction promotes a shift toward aerobic glycolysis (Warburg effect), enhances lipid biosynthesis, and suppresses oxidative phosphorylation, thereby supporting cellular proliferation under hypoxic conditions (117–119).

In addition to *TP53* alterations in NECs, mutations in genes such as *APC, MLL2* (also known as *KMT2D*), and *SF3B1* have also been reported (116). Although these mutations are not currently actionable, they may cooperatively modulate cellular metabolism. For example, APC mutations can activate the Wnt/β-catenin signaling pathway, which increases glucose uptake and stimulates anabolic metabolic programs (120, 121). Mutations in *MLL2*, an epigenetic regulator, and *SF3B1*, a key component of the RNA splicing machinery, have the potential to alter the expression of metabolic enzymes and transporters through epigenomic remodeling or splicing modulation (6, 122, 123).

In another case of a well-differentiated grade 3 NET, the molecular analysis identified additional alterations in PIK3CA, BCL2, and SETD2 in addition to *TP53* mutations (116). In particular, the activating mutation in *PIK3CA* leads to constitutive activation of the PI3K/AKT/mTOR pathway (124), a key regulator of glucose uptake, aerobic glycolysis, nucleotide biosynthesis, and protein synthesis - critical processes for maintaining tumor cell survival and proliferation under metabolic stress (120, 121, 124). In addition, SETD2, a histone methyltransferase, plays a role in regulating gene expression associated with oxidative phosphorylation and the DNA damage response, potentially contributing to a more adaptive cellular phenotype (6, 121, 122). Alterations in BCL2, a key regulator of apoptosis and mitochondrial metabolism, may further impair mitochondrial integrity and promote a glycolytic metabolic shift (125, 126).

In secondary hepatic NENs, elevated levels of urinary 5-hydroxyindoleacetic acid (u5HIAA) - reflecting the secretory activity of functioning tumors - have been associated with increased tumor aggressiveness, disease progression, and the presence of liver metastases (127, 128).

Metabolomic profiling of liver metastases from small intestinal NETs (SI-NETs) has revealed elevated levels of malignancy-associated metabolites - including choline, phosphocholine, taurine, and lactate - compared to normal liver tissue (129). Additional metabolites such as acetate, succinate, and aspartate also contribute to the metabolic signature of metastatic lesions. Acetate is a marker of fatty acid synthesis via acetyl-CoA, while aspartate is considered a limiting metabolite for cell proliferation in cancers with mitochondrial dysfunction. Notably, NET cells appear to be particularly vulnerable to aspartate depletion due to their inherently low asparaginase activity and limited permeability to exogenous aspartate (130).

Furthermore, metabolites such as glucose, alanine, and ethanolamine have been identified as discriminative markers in liver metastases compared to primary SI-NETs (129). Interestingly, these metabolites-along with valine-were found in higher concentrations in normal liver tissue compared to metastatic lesions, highlighting the role of the hepatic microenvironment in shaping the metabolic phenotype of metastatic NETs. These data support the concept of metastatic site-specific metabolic reprogramming and suggest that liver colonization drives a distinct biochemical profile shaped by tumor-host interactions (131).

Accumulating evidence supports the role of epigenetic alterations in the development and progression of NENs. Karpathakis et al. (2017) demonstrated that global DNA hypomethylation is enhanced in liver metastases and is associated with the upregulation of key signaling components including PI3K, EGFR, PDGFRβ, and mTOR (132). Notably, dysregulation of these same pathways has also been reported in primary NENs (133), highlighting shared molecular mechanisms between primary and metastatic disease.

In the broader context of NENs, microRNA-210 (miR-210) has emerged as a critical biomarker linked to tumor progression, metabolic reprogramming, and liver metastasis. Elevated expression of miR-210 and miR-21 correlate with higher Ki-67 proliferation index and the presence of liver metastases in NEs (134) and adenocarcinomas (135), supporting their potential use as prognostic markers. miR-210 is particularly responsive to hypoxic conditions and promotes metabolic adaptation by shifting energy production toward anaerobic glycolysis, one of the hallmarks of malignancy. Its overexpression suppresses tricarboxylic acid (TCA) cycle activity while promoting lactate production, contributing to the Warburg effect (136, 137).

Beyond its role in NETs, miR-210 regulates key targets such as vascular endothelial growth factor (VEGF), a major driver of angiogenesis, further highlighting its potential as a therapeutic target, particularly in tumors where neovascularization and metabolic flexibility are essential for progression (138). The consistent association of miR-210 with metabolic remodeling, cellular stress responses, and tumor aggressiveness positions it as a central molecular integrator of hypoxia-driven tumor adaptation (136, 139, 140).

### 5.7 Gallbladder and bile ducts

To our knowledge, no studies have investigated the role of classical metabolic markers in NENs of this anatomical location. However, emerging evidence points to molecular players that may influence metabolic reprogramming, providing new insights into the gallbladder and bile ducts NENs (12).

Metabolic reprogramming in neoplasia has been increasingly implicated in the activation of oncogenic signaling pathways, adaptation to the tumor microenvironment, and epigenetic regulation. Recent evidence indicates that microRNA-200c (miR-200c) plays a central role in maintaining cholangiocyte homeostasis by suppressing proliferation and neuroendocrine differentiation through inhibition of SESN1 and the IL-6/AKT axis (141). This signaling axis, known for its involvement in the reprogramming of glucose metabolism (i.e., the Warburg effect), also regulates amino acid and nucleotide metabolism via mTORC1 activation (142–145). Dysregulation of SESN1 - a key cellular stress sensor - may further promote adaptive responses such as autophagy and survival under hypoxic conditions, contributing to a more aggressive and metabolically active tumor phenotype, a hallmark of NENs (146, 147).

In the same context, CD117 (c-Kit) expression, although traditionally associated with proliferation in gastrointestinal stromal tumors (GISTs), may also have indirect effects on tumor metabolism. c-Kit is capable of activating the PI3K/AKT/mTOR pathway (133, 148, 149), which has previously been implicated in NEN pathogenesis (133). This activation can enhance glucose uptake, stimulate aerobic glycolysis through the regulation of glucose transporters such as GLUT1, and promote nucleotide and protein biosynthesis - processes essential for rapid cell proliferation (150, 151).

Interestingly, immunohistochemical positivity for CD117 has been reported in a case of MiNEN (large cell neuroendocrine carcinoma and adenocarcinoma) of the gallbladder (152). Although CD117 is not a direct metabolic marker, its expression may reflect the activation of key metabolic pathways, particularly those involved in glucose and nucleotide biosynthesis, thus serving as an indirect indicator of metabolic reprogramming in these rare tumors.

## 5 Conclusion and future directions

Metabolic reprogramming is increasingly recognized as a key feature in the pathogenesis of GEP-NENs. Markers such as GLUT-1, HIF-1α, FASN, LAT-1, and CA9 have been associated with tumor aggressiveness, metastasis, and poor prognosis, particularly in PanNENs. Additionally, epigenetic modulators and hypoxia-induced pathways further contribute to metabolic adaptation and therapeutic resistance.

Despite these insights, metabolic biomarkers have not yet been fully integrated into clinical decision-making. A key limitation of the current literature is the lack of studies directly comparing the metabolic profiles of the various clinical and biological subtypes of GEP-NENs. Tumor functionality, grade, differentiation status, primary site, and hereditary background likely drive distinct metabolic phenotypes; however, available studies do not provide stratified data according to these variables. Consequently, it remains challenging to delineate specific metabolic signatures for these subgroups. Most studies are based on small cohorts and lack external validation, and knowledge regarding nucleotide metabolism, mitochondrial regulation, and amino acid pathways in specific NEN subtypes remains limited. Furthermore, near-term research priorities include: (1) multicenter validation of GLUT-1, FASN, and CA9 cut-offs using standardized IHC; (2) prospective studies linking dual-tracer PET imaging to tissue metabolism and outcomes; (3) development of plasma and urine metabolomics panels benchmarked against CgA and u-5HIAA; (4) trials evaluating metabolic co-targeting strategies, such as mTOR inhibition combined with lipogenesis or ferroptosis modulation; (5) investigations designed to explore metabolic heterogeneity in NENs. These approaches aim to translate metabolic insights into clinically actionable tools and address the heterogeneity of GEP-NENs.
